# Supplementary material for: Prevalence of Depression and Predictors of Discharge to a Psychiatric Hospital in Young People with Hospital-Treated Deliberate Self-Poisoning at an Australian Sentinel Unit
Source: Int J Environ Res Public Health. 2022 Nov 26;19(23):15753. doi: 10.3390/ijerph192315753 (PMC9737120; doi:10.3390/ijerph192315753)
Supplement: Supplementary file 1 [file ijerph-19-15753-s001.zip › ijerph-2030071-supplementary.pdf]

Supplementary Table S1: Univariate demographic characteristics predicting discharge destination (referral to psychiatric hospital vs. other).

Categorical		Psychiatric hospital[1] N = 358 n (%)	Other[2] N = 1052 n (%)	Total N = 1410 n (%)	Chi square	p value
Gender	Male	134 (37.4)	353 (33.6)	487 (34.5)	1.77	0.183
	Female	224 (62.6)	699 (66.4)	923 (65.5)		
Cultural background	Indigenous	26 (7.3)	117 (11.1)	143 (10.1)	4.37	0.037
	Non-Indigenous	332 (92.7)	935 (88.9)	1267 (89.9)		
Marital status *(missing = 2)*	Single/never married	303 (84.6)	881 (83.8)	1184 (84.0)	0.60	0.740
	Married/de facto	36 (10.1)	121 (11.5)	157 (11.1)		
	No longer partnered[3]	18 (5.0)	49 (4.6)	67 (4.8)		
Employment	Employed/Student	185 (51.7)	559 (53.1)	744 (52.8)	2.47	0.291
	Not working/studying	143 (39.9)	381 (36.2)	524 (37.2)		
	Unknown	30 (8.4)	112 (10.6)	142 (10.1)		
Highest Education	Primary School	18 (5.0)	60 (5.7)	78 (5.5)	60.52	<0.001
	High School	264 (73.7)	797 (75.8)	1061 (75.3)		
	Tertiary Education	52 (14.5)	135 (12.8)	187 (13.3)		
	Unknown	24 (6.7)	60 (5.7)	84 (6.0)		
Housing	Secure	325 (90.8)	978 (93.0)	1303 (92.4)	8.31	0.016
	Insecure	23 (6.4)	33 (3.1)	56 (4.0)		
	Unknown	10 (2.8)	41 (3.9)	51 (3.6)		
Referral Area	Primary	261 (72.9)	787 (74.8)	1048 (74.3)	0.63	0.730
	Secondary	82 (22.9)	220 (20.9)	302 (21.4)		
	Not in Area	15 (4.2)	45 (4.3)	60 (4.3)		
Socio-economic Quintile (SEIFA) *(missing = 17)*	Lowest (quintile 1)	60 (16.8)	193 (18.3)	253 (17.9)	4.66	0.097
	Middle (quintiles 2-4)	270 (75.4)	801 (76.1)	1071 (76.0)		
	Highest (quintile 5)	25 (7.0)	44 (4.2)	69 (4.9)		

Continuous	Psychiatric hospital[1] Mean (SD)	Other[2] Mean (SD)	Total Mean (SD)	t-test	p value
Age on admission (years)	20.56 (2.66)	19.91 (2.56)	20.07 (2.60)	-4.061	<0.001
Length of stay (hours)	30.57 (44.72)	18.35 (16.84)	21.45 (27.32)	-7.450	<0.001

[1] Referral for admission to a psychiatric hospital – private or public
[2] Discharged to place of usual residence/home or police custody or self-discharged
[3] Due to divorce, separation, or death

Supplementary Table S2: Univariate Clinical characteristics predicting discharge destination (referral to psychiatric hospital vs. other).

Characteristics		Psychiatric hospital[1] N = 358 n (%)	Other[2] N = 1052 n (%)	Total N =1410 n (%)	Chi square	p value
Diagnostic Categories (DSM)[3,4]	Any Depression	207 (57.8)	293 (27.9)	500 (35.5)	94.67	<0.001
	Depressive Disorder	194 (54.2)	275 (26.1)	469 (33.3)	4.58	0.032
	Bipolar Disorder	13 (3.6)	18 (1.7)	31 (2.2)	104.83	<0.001
	Any Anxiety	59 (16.5)	152 (14.4)	211 (15.0)	0.87	0.352
	Any Psychosis	13 (3.6)	13 (1.2)	26 (1.8)	8.47	0.004
	Any Substance Use	118 (33.0)	326 (31.0)	444 (31.5)	0.482	0.488
	Relational Problem	106 (29.6)	447 (42.5)	553 (39.2)	18.59	<0.001
	Personality Disorder	124 (34.6)	343 (32.6)	467 (33.1)	0.498	0.480
	Other	157 (43.9)	639 (60.7)	796 (56.5)	30.98	<0.001
Suicidal Level	None	56 (15.6)	885 (84.1)	941 (66.7)	666.51	<0.001
	Low/Moderate	130 (36.3)	126 (12.0)	256 (18.2)		
	High	158 (44.1)	17 (1.6)	175 (12.4)		
	Unknown	14 (3.9)	24 (2.3)	38 (2.7)		
Previous self-harm	Absent	113 (31.6)	410 (39.0)	523 (37.1)	7.72	0.005
	Present	245 (68.4)	642 (61.0)	887 (62.9)		
Psychiatric Treatment (past 12 months)	No contact	94 (26.3)	381 (36.2)	475 (33.7)	27.79	<0.001
	Outpatient only	164 (45.8)	502 (47.7)	666 (47.2)		
	Inpatient	100 (27.9)	169 (16.1)	269 (19.1)		
Life Events (past month) *(missing = 19)*	None	142 (39.7)	350 (33.2)	492 (34.9)	5.016	0.081
	1	117 (32.7)	397 (37.7)	514 (36.5)		
	2 or more	96 (26.8)	289 (27.5)	385 (27.3)		
Presentation time	In hours	92 (25.7)	214 (20.3)	306 (21.7)	4.51	0.034
	Out of hours	266 (74.3)	838 (79.7)	1104 (78.3)		

	Psychiatric hospital[1] Mean (SD)	Other[2] Mean (SD)	Total Mean (SD)	t-test	p-value
No. of diagnoses *(missing 6)*	1.62 (0.84)	1.23 (0.81)	1.33 (0.83)	-7.847	<0.001
No. support persons *(missing 211)*	2.55 (2.78)	3.13 (1.90)	2.98 (2.18)	3.406	0.001
No. life events (month) *(missing 19)*	1.04 (1.14)	1.07 (1.03)	1.06 (1.06)	0.423	0.672

[1]: Referral for admission to a psychiatric hospital – private or public
[2]: Discharged to place of usual residence/home or police custody or absconded
[3]: Diagnostic and Statistical Manual (DSM IV or DSM 5 depending on coding used at presentation) [4]: More than 1 diagnosis is possible
